# Various Techniques and Outcomes of Arterial Anastomosis in Live Renal Transplant: An Institutional Experience

**DOI:** 10.7759/cureus.25262

**Published:** 2022-05-23

**Authors:** Brijesh Tiwari, Pranchil Pandey, Vezhaventhen G, Saravanan K

**Affiliations:** 1 Urology, Shyam Shah Medical College, Rewa, IND; 2 Anaesthesiology, Shyam Shah Medical College, Rewa, IND; 3 Urology, Madras Medical College, Chennai, IND

**Keywords:** vessel anastomosis, kidney transplant, transplant renal artery stenosis, multiple renal artery, living related renal transplant

## Abstract

Introduction

Renal transplantation with multiple arteries is associated with a major index of surgical complications. Relevant papers and meta-analyses have shown relatively more vascular and urological complications in transplant of donor kidneys with multiple arteries. In live donor grafts due to the unavailability of a carrel patch, several techniques for bench and in situ reconstruction have been described in order to reduce the incidence of these vascular complications. In this study, the short and long-term results of living donor kidney transplants with multiple renal arteries (MRAs) versus single renal artery (SRA) were compared retrospectively.

Methods

This is a retrospective study done on patients who received a living donor kidney between January 2012 and January 2018 at* *the* *Institute of Urology*,* Madras Medical College, Chennai. We have excluded deceased donor kidney transplants and ABO-incompatible cases done in the same time period. The study was approved by the Institutional Ethics Committee (Approval No: IES-MMC-008) and performed in accordance with the guidelines of the Declaration of Helsinki. Open live donor nephrectomy was performed through an extra-peritoneal flank incision in all cases. In the SRA group, the renal artery was anastomosed end to end to the Internal iliac artery, while the renal vein was anastomosed to the external iliac vein in the end to side fashion. Urinary tract reconstruction was accomplished by the Gregoir technique in both groups. We looked at recipient complications, baseline and postoperative serum creatinine, total ischemia time, mean operating time, and short- and long-term graft and patient survival as postoperative outcomes.

Results

In a six-year period (2012-2018) at our institute, 256 living donor transplantations were performed; 36 (14%) kidneys had two or more renal arteries which were anastomosed using various techniques. Cold ischemia time was relatively longer in the MRA group (45 mins vs 28 mins in the SRA group) (p-value <0.05). while warm ischemia time was comparable in both groups (2.5 vs 2.9 mins) serum creatinine was comparable in both groups at the 30th postoperative day (1.4 in SRA group vs 1.2 in MRA group) (p-value >0.05). Incidence of surgical complications in SRA and MRA groups was: vascular - 3.6% and 2.7%; urological - 3.2% and 2.7%; the incidence of lymphocele was 4.5% and 5.5% and delayed graft function 4.5% and 5.5% respectively.

Conclusion

Multiple renal arteries are no longer a relative contraindication with advanced surgical techniques. in renal grafts with multiple arteries, all techniques of vessel anastomosis are comparable in terms of post-surgical complications.

## Introduction

The occurrence of multiple renal vessels is common in the general population. Thus, vascular anomalies in live-related renal donors are a common presentation. Renal transplantation with multiple arteries has been associated with a theoretically higher rate of surgical complications. In view of the limited donor pool, transplants of grafts with multiple vessels are being done electively. The standard approach for renal transplant with a single artery is technically easier because of the single ostium. But in graft with multiple arteries, either a carrel patch or a bench procedure is needed before proceeding with anastomosis. In grafts from cadaver donors, the carrel aortic patch is the standard technique of vascular reconstruction but for live donor grafts, due to the unavailability of a carrel patch, several techniques for bench and in situ reconstruction have been described in order to reduce the incidence of these vascular complications [1].

Our primary objective was to highlight, various techniques of arterial anastomosis performed for renal transplants in kidneys with multiple vessels and their surgical outcome. In this study, the short- and long-term results of living donor kidney transplants with multiple renal arteries (MRAs) versus single renal artery (SRA) were compared retrospectively.

## Materials and methods

This is a retrospective study done on patients who received a living donor kidney between January 2012 and January 2018 at the Institute of Urology, Madras Medical College, Chennai. We have excluded deceased donor kidney transplants and ABO-incompatible cases done in the same time period. The study was approved by the Institutional Ethics Committee (Approval No: IES-MMC-008) and performed in accordance with the guidelines of the Declaration of Helsinki.

Detailed clinical history was recorded, and physical examination was performed on the recipients. The donor's renal vascular anomalies were assessed using computed tomography angiography. Before the transplant, human leukocyte antigen typing, and tissue crossmatch between donors and recipients were done. ABO blood group compatibility was assessed on all donors and recipients.

Open live donor nephrectomy was performed through an extraperitoneal flank incision in all the cases. The left kidney was preferred over the right for donor nephrectomy, except in cases of vascular problems or any other contraindications for which the right kidney was preferred. In all cases, kidneys were placed in the right iliac fossa for implantation by an extraperitoneal approach. In the SRA group renal artery was anastomosed end to end to the internal iliac artery, while the renal vein was anastomosed to the external iliac vein in the end to side fashion. The techniques applied for arterial reconstructions in the MRA group are presented in Table 1. Fine polypropylene sutures (Prolene 6-0; Ethicon LLC, Puerto Rico, United States) were used for vascular anastomoses. For smaller caliber arteries, Proline 7-0 sutures (Ethicon LLC, Puerto Rico, United States) were used for anastomosis. Urinary tract reconstruction was accomplished by the Gregoir technique in both groups. A double J stent was not routinely placed during ureteroneocystostomy. A double J stent was only inserted in select patients with neurogenic bladders, ureteral abnormalities, small stones in the donor's kidneys, and reduced or no immediate urine output after the vascular anastomosis. The drain was removed if it was not draining for 24 hours, and the urethral catheter was removed usually on the fifth postoperative day. Doppler ultrasonography was routinely performed on the third day after transplant to evaluate vascular flow as well as the urinary system. CT angiogram was done in suspected cases of arterial stenosis. CT cystogram was done in suspected cases of urological complications.

**Table 1:** Vascular technique in renal grafts with multiple arteries IIA: Internal iliac artery; EIA: External iliac artery

Intraoperative Procedure	Bench Procedure	N=36	%
End to end anastomosis of main/re-constructed renal artery to IIA	Superior polar artery ligature	12	33.3%
	Side to side anastomosis of 2 arteries (double barrel) (Wallace technique)	12	33.3%
	End to side anastomosis of lower polar artery to main renal artery	06	16.6%
End to end anastomosis of upper polar to IIA and end to side anastomosis of lower polar to EIA		04	11.1%
End to end anastomosis of the upper polar artery to IIA and end to side anastomosis of the lower polar artery to EIA (triple vessel)	Superior polar artery ligation	02	5.5%

All recipients were given prophylactic broad-spectrum parenteral antibiotics in the operating room during the transplant along with optimal hydration. Recipients were given 500 mg and 125 mg methylprednisolone intraoperatively in the evening on the day of the transplant. Furthermore, these patients received 20 mg of prednisolone for the first month, 15 mg for the second month, and 10 mg after the third month. Tacrolimus was given at a dose of 0.12 mg/kg/day, and we monitored tacrolimus serum levels to avoid toxicity. Mycophenolate mofetil was started at a dose of 2 g for three months and was given at a dose of 1.5 g thereafter. We used cotrimoxazole and valganciclovir for prophylaxis. We looked at recipient complications, baseline and postoperative serum creatinine, total ischemia time, mean operating time, and short- and long-term graft and patient survival as postoperative outcomes.

We used Kaplan-Meier analysis to compute the survival function. Qualitative data are presented as frequencies and percentages. Mean values (with Standard Deviation) were calculated for quantitative variables. Statistical analysis was done using the chi-square test, Fisher exact test, and the non-parametric Kruskal Wallis test. A value of p < 0.05 was considered statistically significant. Data analysis was carried out by using Statistical Package for the Social Sciences (SPSS) Version 20.0 (IBM Corp., Armonk, NY).

## Results

A total of 256 living donor transplantations were performed. Patients were divided into two groups: SRA group - grafts with a single artery (220 patients) and MRA group - grafts with multiple arteries (36 patients). In the MRA group, 34 kidneys had two renal arteries and two had three renal arteries. Preoperative mean serum creatinine was comparable in both groups (4.1 vs 4.3) (Table 2).

**Table 2:** Demographic data and preoperative characteristics SRA: Single renal artery; MRA: Multiple renal arteries

	SRA group (220)	MRA group (36)	p-value
Male/ Female	134/86	24/12	
Median age	42.1	43.6	0.546
Mean serum creatinine	4.1	4.3	0.123
Arteries per graft	1	34 cases- 2 arteries 2 cases - 3 arteries	

Cold ischemia time was relatively longer in transplants with multiple arteries (45 mins vs 28 mins in single renal arteries) (p-value <0.05). Warm ischemia time was comparable in both groups (2.5 vs 2.9 mins). Serum creatinine was comparable in both groups on the 30th postoperative day (1.4 in SRA group vs 1.2 in MRA group) (p-value >0.05) (Table 3).

**Table 3:** Intraoperative and postoperative variables SRA: Single renal artery; MRA: Multiple renal arteries

	SRA group (n=220)	MRA group (n=36)	p-value
Cold ischemia (Minutes)	28	45	< .05>
Warm ischemia (Minutes)	2.5	2.9	>.05
Serum creatinine (Postoperative day 30)	1.4	1.2	>.05

The incidence of surgical complications in grafts with SRA and MRA groups were: vascular - 3.6% and 2.7%; urological - 3.2% and 2.7% and the incidence of lymphocele was 4.5% and 5.5% and Delayed graft function 4.5% and 5.5% respectively. Renal graft losses were due to arterial thrombosis and occurred in six (2.7 %) grafts with a single artery, no arterial thrombosis, and graft loss was reported in grafts with multiple arteries (Table 4).

**Table 4:** Complications SRA: Single renal artery; MRA: Multiple renal arteries

Complications		SRA group (n=220)	MRA group (n=36)	p-value
Vascular complications	Arterial stenosis	8(3.6%)	1(2.7%)	>.05
	Arterial thrombosis	6 (2.7%)	0	>.05
Urologic complications	Urinary leak /fistula	5(2.2%)	1(2.7%)	>.05
	Ureteral stenosis	2(1%)	0	>.05
Lypmhocele		10 (4.5%)	2 (5.5%)	>.05
Delayed graft function		10(4.5%)	2(5.5%)	>.05

There was no difference in postoperative complications among various vascular techniques of the MRA group. Only one case of arterial stenosis was reported in the group of end to side anastomosis of the lower polar artery to the main renal artery. Two cases of urinary fistula were reported which were managed by revision of ureteroneocystostomy and two cases of postoperative lymphocele were reported, managed conservatively. All complications were in insignificant numbers and none attributed to any single type of vessel anastomosis (Table 5).

**Table 5:** Complications among various techniques of anastomosis in multiple renal artery grafts IIA: Internal iliac artery; EIA: External iliac artery

Intraoperative Procedure	Bench Procedure	Arterial Stenosis	Arterial Thrombosis	Lymphocele	Urologic Complication
End to end anastomosis of the reconstructed renal vessel to IIA	Superior polar artery ligature	-	-		
	Side to side anastomosis of 2 arteries (double barrel) (Wallace technique)	-	-		1
	End to side anastomosis of lower polar artery to main renal artery	1	-	1	
End to end anastomosis of upper polar to IIA and end to side anastomosis of lower polar to EIA			-		1
End to end anastomosis of upper polar to IIA and end to side anastomosis of lower polar to EIA (triple vessel)	Superior polar artery ligation	-	-	1	

Our results suggest that there is only a theoretical risk of complications present in the transplant of multiple renal arteries, as apart from cold ischemia time all complications were comparable in both groups.

## Discussion

Graft with multiple renal arteries has been associated with a higher rate of vascular complications, including arterial thrombosis and renal artery stenosis [2]. At the beginning of the renal transplantation era, this fact was considered a contraindication to the procedure. In our study, we reviewed the incidence of surgical complications in transplants with a single artery and transplants with multiple arteries and compared them with each other. 

MRAs are a common anatomic variant of the kidney. In the present study, the incidence of MRAs was found to be 16.36%, which is lower than the value of 30% reported by Aydin and colleagues (2004) [3]. Arterial stenosis occurs in a range of 0.8 to 12.4% of all renal transplants [1]. In our study, there were nine cases (3.5%) of renal arterial stenosis, most of them in grafts with a single artery. In the MRA group, only one case of arterial stenosis was reported. Most of them were managed conservatively. Revascularization (resection of stenotic portion and re-anastomosis) has been done in two cases of the SRA group in which arterial stenosis caused declining renal function and obstruction was greater than 50%. 

Postoperative outcomes, such as acute rejection, creatinine levels, surgical complications, and graft survival, were comparable across the two groups in this study. Similar findings have been found in a number of previous studies [4]. In terms of vascular and urologic complications, as well as patient and graft survival rates, Ashraf and colleagues (2013) concluded that allograft kidney transplants with MRAs are equally safe and successful as transplants with SRA [5]. Delayed graft function was found in a total of 12 cases. Incidence was slightly higher in the MRA group (5.5% vs 4.5 %in the single renal artery group), but it was statistically insignificant, and this fact can be explained by the manipulation necessary for vascular reconstruction in these grafts. Previously published studies reported frequent incidence of lymphoceles (1%-12%) with MRAs [6]. We have found an almost similar incidence of lymphocele in grafts with multiple arteries (5.5%) compared to grafts with a single artery (4.5%), (p >0.05). Usually, the lymphatic vessels are more abundant in grafts with multiple arteries and so they are vulnerable to insufficient ligature explaining the higher incidence of lymphocele in this group.

Delayed graft function was found in a total of 12 cases. Incidence was slightly higher in the MRA group (5.5% vs 4.5 % in the single renal artery group), but it was statistically insignificant, and this fact can be explained by the manipulation necessary for vascular reconstruction in these grafts. Renal arterial thrombosis caused graft loss in six patients, all of them with a single artery. In our series, we did not find any arterial thrombosis in grafts with multiple arteries.

The current study has several limitations, including a limited sample size and the study being conducted at a single center only. Also, given the type of population plays a big role in kidney transplant outcomes, extrapolating these findings to a larger population of people of other ethnicities is not appropriate.

## Conclusions

With the advancement of surgical techniques, multiple renal arteries are no longer a relative contraindication. In terms of complications and long-term graft and patient survival, all techniques of vascular anastomosis are comparable among MRA grafts.

## Appendices

Cold Ischemia Time (CIT): It is defined as the interval from initiation of the donor in vivo cold organ preservation to the removal of the graft from 4°C cold storage. Typically, 4-6 hours for a heart, < 12 hours for liver and pancreas, and < 24 hours for a kidney. Shortening the CIT may reduce the risk of post-transplantation graft failure and patient mortality, and longer hospital stay.

Warm Ischemia Time (WIT): It is defined as the interval from circulatory arrest to the removal of organs and initiation of cold organ preservation. This is typically 20-30 minutes (<60 minutes).
